# The necessity of abdominal drainage for patients with complicated appendicitis undergoing laparoscopic appendectomy: a retrospective cohort study

**DOI:** 10.1186/s13017-022-00421-3

**Published:** 2022-03-17

**Authors:** Yu-Tso Liao, John Huang, Chia-Tung Wu, Pei-Chen Chen, Tsung-Ting Hsieh, Feipei Lai, Tzu-Chun Chen, Jin-Tung Liang

**Affiliations:** 1 Graduate Institute of Clinical Medicine, College of Medicine, National Taiwan University, Taipei, Taiwan, ROC; 2 Division of Colorectal Surgery, Department of Surgery, National Taiwan University Hospital, Hsin-Chu Branch, Hsinchu, Taiwan, ROC; 3 Division of Colorectal Surgery, Department of Surgery, National Taiwan University Hospital and College of Medicine, Taipei, Taiwan, ROC; 4 Department of Computer Science and Information Engineering, National Taiwan University, Taipei, Taiwan, ROC; 5 Graduate Institute of Biomedical Electronics and Bioinformatics, National Taiwan University, Taipei, Taiwan, ROC

## Abstract

**Background:**

This study aimed to evaluate the necessity of abdominal drainage after laparoscopic appendectomy in patients with complicated appendicitis.

**Methods:**

Patients with acute appendicitis undergoing laparoscopic appendectomy at two hospitals between January 2014 and December 2018 were retrospectively included. Complicated appendicitis was defined as the American Association for the Surgery of Trauma (AAST) grade ≥ II. The patients were classified according to the AAST grade and the indwelling of abdominal drainage. The postoperative surgical outcomes and recovery were compared among patient groups to evaluate the impact of abdominal drainage for patients with complicated appendicitis undergoing laparoscopic appendectomy.

**Results:**

A total of 1241 patients was retrospectively included. Among them, there were 820 patients with simple appendicitis (AAST grade I) and 421 patients with complicated appendicitis (AAST grade ≥ II). For complicated appendicitis, the drainage group (*N* = 192) tended to harbor more overall complications, intra-abdominal abscess formation, time to resume a soft diet, and the postoperative length of hospitalization (*P* = 0.0000 for all). Multivariate logistic regression confirmed that abdominal drainage increased the risk of overall complications [Odds ratio (OR) 2.439; 95% confidence interval (CI) 1.597–3.726; *P* ≤ 0.0001] and failed to decrease the risk of intra-abdominal abscess formation (OR 1.655; 95% CI 0.487–5.616; *P* = 0.4193). Multivariate linear regression analysis also showed that the drainage group harbored longer postoperative length of hospitalization (Coefficients: 20.697; 95% CI 15.251–26.143; *P* < 0.0001) and time to resume a soft diet (Coefficients: 45.899; 95% CI 34.502–57.297; *P* < 0.0001).

**Conclusions:**

Abdominal drainage did not prevent overall complications in patients with complicated appendicitis; paradoxically, it delayed the convalescence. Our results discourage the routine use of abdominal drainage and suggest that abdominal drainage should be performed sparingly.

## Background

Acute complicated appendicitis is a common disease that usually requires emergency surgery. The necessity of abdominal drainage is considered for monitoring and preventing postoperative intra-abdominal abscess (IAA) formation, especially for perforated appendicitis with general peritonitis. Although many primary studies and subsequent systematic reviews and meta-analyses have investigated this field of research in the past, and at least three Cochrane Reviews have been published showing that "there is no evidence for any clinical improvement with the use of abdominal drainage in patients undergoing open appendectomy for complicated appendicitis", quality of evidence is still low and deserves further research [1–9]. Laparoscopic appendectomy (LA) has recently become the mainstay of surgical method for treating acute appendicitis. Several retrospective studies have questioned abdominal drainage for the prevention of IAA after LA [5, 6, 9–11]. However, there is no consensus regarding the necessity of abdominal drainage after LA [12].

The American Association for the Surgery of Trauma (AAST) grade for acute appendicitis has been widely adopted for predicting complications, recovery, and hospital costs [13, 14]. Complicated appendicitis is defined based on the severity of inflammation as follows: grade II (gangrenous appendix), III (perforated appendix with focal contamination), IV (perforation appendix with abscess formation), and V (perforated appendix with generalized peritonitis). For simplicity, most studies defined the severity of appendicitis by dichotomizing it into simple vs. complicated appendicitis[8–10, 15] or using descriptive terms such as gangrenous, perforated, or peritonitis [6, 11]. One study used the AAST grading system to evaluate the association between abdominal drainage and postoperative IAA after LA [7].

In the present study, we aimed to evaluate the necessity of abdominal drainage after LA by analyzing patients with complicated appendicitis, who were classified according to AAST grade.

## Materials and methods

### Patient selection

A total of 1241 patients diagnosed with acute appendicitis and undergoing LA at the Yunlin (*N* = 533) and Hsinchu (*N* = 708) branch hospital of National Taiwan University Hospital were included for the present study between January 2014 and December 2018. The diagnosis of appendicitis was based on medical history, physical examination, imaging studies including abdominopelvic computed tomography (CT), and histopathological examination of surgical specimens. The AAST grade system was used to determine the severity of appendicitis [16]. Complicated appendicitis was defined as AAST grade ≥ II.

### Determination of AAST grade

**Fig. 1:**
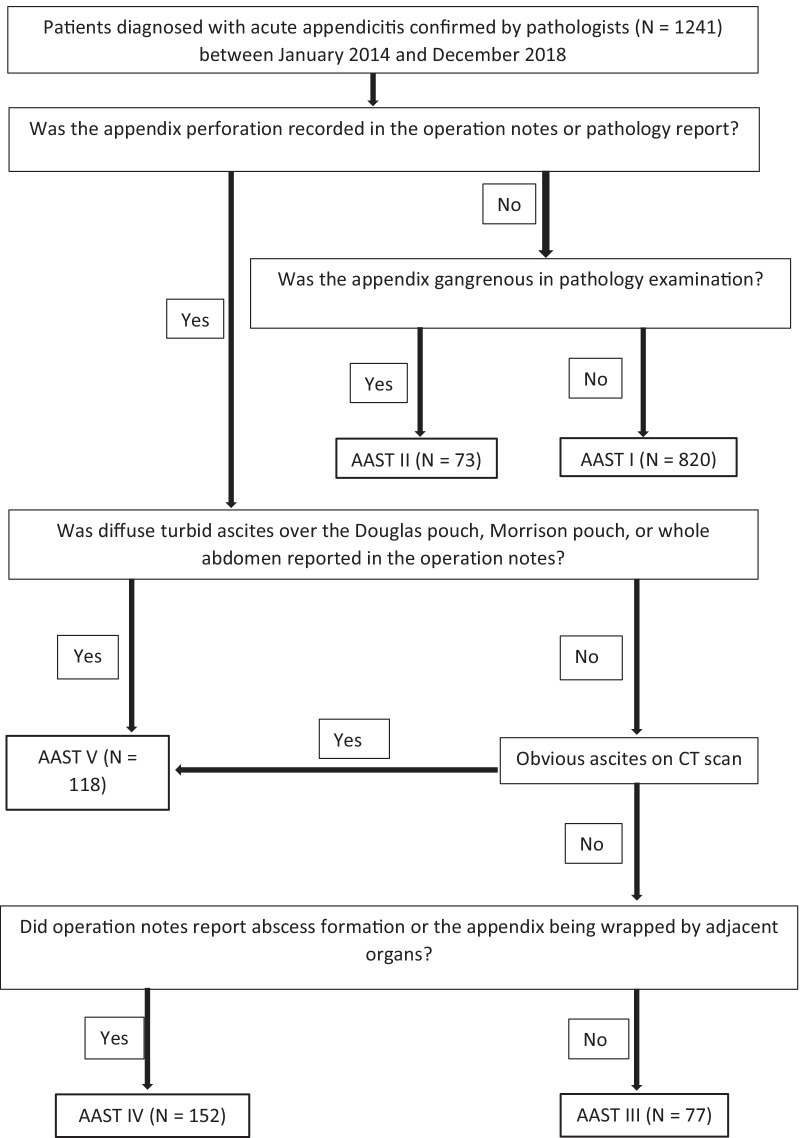
The algorithm for patient allocation according to AAST grade

An algorithm was developed to determine the AAST grade for each patient (Fig. 1). The determination of AAST grade for each patient was based on operation notes and pathology and radiology reports. The AAST grade of all patients was determined by the author (Dr. Yu-Tso Liao). This study was approved by the ethical committee of National Taiwan University Hospital (202104032RINB). Informed consents were waived because of the retrospective nature of the study and the analysis used anonymous clinical data.

### Evaluation of surgical outcomes

The primary endpoint for this study was surgical complications within 30 days after LA. The Clavien–Dindo classification system was used to determine the severity of surgical complications, including wound infection, postoperative ileus, and IAA formation. IAA formation was defined as abdominal abscess formation observed on ultrasound or CT within 30 days after LA. Surgical site infection was defined as clinical pus formation or erythematous change of the wound, requiring antibiotic treatment within 30 days after LA. Postoperative ileus was defined by symptoms and signs of abdominal distention, nausea or vomiting, followed by confirmation based on serial abdominal radiography such as plain X-ray or CT within 30 days after LA.

The secondary endpoint was postoperative recovery, which was evaluated according to the time to resume a soft diet and the postoperative length of hospitalization (LOH). Postoperative LOH, the duration between leaving the recovery room and the time of discharge, was recorded in hours. Operative time was defined as the time from skin incision to the application of the wound dressing.

### Postoperative care

Patients with simple appendicitis were allowed to resume a soft diet immediately after surgery. For patients with complicated appendicitis, a diet limited to a small amount of clear liquid was initiated. Permission to resume a soft diet was given to the patients after flatus and the absence of abdominal distention. The criterion for drain removal was serosanguinous fluid < 50 mL/day.

### Statistical methods

Statistical analyses between two groups were performed using the independent *t*-test, the chi-square test, and Fisher’s exact test. Multivariate logistic regression analysis with stepwise selection was used to identify independent prognostic factors associated with categorized variables, such as complications. Multivariate linear regression was used for continuous variables, including the time to resume a soft diet and postoperative LOH. Statistical significance was set at *P* < 0.05. All analyses were performed using SAS version 9.4 for Windows (SAS Institute Inc., Cary, NC, USA).

## Results

A total of 1241 patients who underwent LA between January 2014 and December 2018 were included in our study. The clinical presentations on arrival at the emergency department are shown in Table 1.

**Table 1:** Clinical characteristics of patients with simple appendicitis (AAST grade I) and complicated appendicitis (AAST grade ≥ II) classified by indwelling abdominal drainage status

	Simple appendicitis (AAST I, *n* = 820)			Complicated appendicitis (AAST II/III/IV/V, *n* = 421)			
	+ drainage (*N* = 82)	− drainage (*N* = 738)	*P* value	+ drainage (*N* = 192)	− drainage (*N* = 229)	*P* value	*P* value^1^
Age, yrs (mean ± SD)	41.27 ± 18.79	38.12 ± 17.15	0.1183	46.38 ± 19.01	42.44 ± 19.93	0.0395	** < 0.0001**
*Sex, n*			0.3157			0.1179	0.0092
Female (%)	34 (41.46)	349 (47.29)		67 (34.90%)	97 (42.36%)		
Male (%)	48 (58.54)	389 (52.71)		125 (65.10%)	132 (57.64%)		
BMI, kg/m^2^, (mean ± SD)	23.37 ± 3.62	23.45 ± 4.11	0.8741	24.08 ± 4.55	23.57 ± 4.37	0.2370	0.1631
*Pain duration, n (%)*			0.2186			0.0022	< 0.0001
< 24hs	24 (29.27)	171 (23.17)		105 (54.69)	91 (39.74)		
≥ 24 h	58 (70.73)	567 (76.83)		87 (45.31)	138 (60.26)		
*ASA*^1^ *class, n*			0.1102			0.0964	**0.0007**
I/II/III/IV	40/39/3/0	282/398/58/0		55 /105/29/3	83/124/21/1		
Previous abdominal surgery, n(%)	6 (7.32)	71 (9.62)	0.4975	9 (4.69)	29 (12.66)	0.0044	0.8341
Alvarado score (mean ± SD)	5.30 ± 1.75	5.70 ± 1.55	0.0322	6.04 ± 1.59	7.05 ± 7.01	0.0351	0.0005
*AAST grade, n(%)*			NA			0.0012	NA
II (%)	NA	NA		21 (10.94)	52 (22.71)		
III (%)				31 (16.15)	46 (20.09)		
IV (%)				72 (37.50)	80 (34.93)		
V (%)				67 (35.42)	51 (22.27)		

^1^ *P* value was calculated between simple appendicitis (AAST I) and complicated appendicitis (AAST II/III/IV/V)

AAST, The American Association for the Surgery of Trauma; BMI, body mass index; ASA, The American Society of Anesthesiologists

Continuous data were presented with mean ± standard deviation (SD)

In the simple appendicitis group, patients with drainage had a significantly higher Clavien–Dindo classification, longer time to resume a soft diet, longer postoperative LOH, and higher readmission rate than patients without drainage (Table 2).

**Table 2:** Surgical outcomes for patients with simple appendicitis (AAST grade I) and complicated appendicitis (AAST grade ≥ II) classified by indwelling abdominal drainage status

	Simple appendicitis (AAST I, n = 820)			Complicated appendicitis (AAST II/III/IV/V, n = 421)			
	+ drainage (*N* = 82)	− drainage (*N* = 738)	*P* value	+ drainage (*N* = 192)	− drainage (*N* = 229)	*P* value	*P* value^1^
Surgical time, mins, [median (Q1, Q3)]	44.00 (35, 59)	45.00 (30, 57)	0.1190	65.00 (50, 80)	60.00 (50, 91.5)	0.0357	< 0.0001
*Method of stump closure, n (%)*			< 0.0001			< 0.0001	< 0.0001
Endostapler	6 (7.32)	54 (7.32)		26 (13.54)	72 (31.44)		
Endoloop (SurgiTie®)	45 (54.88)	193 (26.15)		81 (42.19)	39 (17.03)		
Hem-o-lok® clips	23 (28.05)	456 (61.79)		66 (34.38)	105 (45.85)		
Intracorporeal suture	4 (4.88)	14 (1.90)		15 (7.81)	8 (3.49)		
Endoclips	4 (4.88)	21 (2.85)		4 (2.08)	5 (2.18)		
*The Clavien–Dindo Classification of complications, n (%)*			0.0431			0.9960	0.1465
I/II	6 (85.71)	21 (95.45)		55 (82.09)	32 (82.05)		
III/IV	1 (14.29)	1 (4.55)		12 (17.91)	7 (17.95)		
Complications Wound infection, n (%)	12 (14.63)	43 (5.83)	0.0025	26 (13.54)	26 (11.35)	0.4968	0.0008
Intra-abdominal abscess, n (%)	1 (1.22)	0 (0.00)	0.1000	7 (3.65)	5 (2.18)	0.3941	< 0.0001
Ileus, n (%)	2 (2.44)	3 (0.41)	0.0809	29 (15.10)	10 (4.37)	0.0002	< 0.0001
Time to resume a soft diet, hrs [median (Q1, Q3)]	20.38 (10.70, 24.07)	16.25 (11.73, 39.85)	0.0044	46.95 (13.08, 38.48)	22.07 (20.00, 81.25)	< 0.0001	**< 0.0001**
Postoperative hospitalization, hrs [median (Q1, Q3)]	63.24 (38.33,63.58)	52.41 (47.60, 103.13)	< 0.0001	115.65 (52.12, 98.85)	68.42 (81.29, 161.45)	< 0.0001	< 0.0001
Readmission, n (%)	3 (3.66)	4 (0.54)	0.0251	4 (2.08)	4 (1.75)	> 0.9999	0.1102

^1^ *P* value was calculated between simple appendicitis (AAST I) and complicated appendicitis (AAST II/III/IV/V)

AAST, The American Association for the Surgery of Trauma

In the complicated appendicitis group (AAST grade ≥ II), patient with drainage were significantly older, had a longer pain duration, and a higher American Society of Anesthesia score (Table 1). In addition, patients with drainage had a longer time to resume a soft diet and longer postoperative LOHs than patients without drainage. Moreover, patients with drainage did not have a lower rate of IAA formation compared to patients without drainage (Table 2).

Multivariate logistic regression analysis for complications showed that abdominal drainage was an independent factor for increased overall complications, wound infection, and ileus. The AAST grade was also an independent factor for increased overall complications, IAA, and ileus (Table 3).

**Table 3:** Multivariate logistic regression analysis for complications

	All complications (Clavien–Dindo classification)		Wound infection		Intra-abdominal abscess		Ileus	
	OR (95% CI)	*P* value	OR (95% CI)	*P* value	OR (95% CI)	*P* value	OR (95% CI)	*P* value
*Drainage*		< 0.0001		0.0309		0.4193		0.0004
No	1.0		1.0		1.0		1.0	
Yes	2.439 (1.597–3.726)		1.692 (1.049–2.729)		1.655 (0.487–5.616)		3.726 (1.788–7.764)	
*Sex*		0.1465		0.1383		0.1648		0.0755
Female	1.0		1.0		1.0		1.0	
Male	1.351 (0.900–2.029)		1.370 (0.903–2.079)		2.555 (0.680–9.598)		1.887 (0.937–3.800)	
Age, yrs	1.020 (1.010–1.031)	0.0002	1.005 (0.994–1.016)	0.3389	1.015 (0.985–1.045)	0.3301	1.026 (1.009–1.044)	0.0033
AAST grade	1.673 (1.469–1.905)	< 0.0001	1.110 (0.962–1.281)	0.1547	2.219 (1.397–3.523)	0.0007	1.693 (1.343–2.135)	**< 0.0001**

OR, odd ratio; CI, confidence interval; AAST, The American Association for the Surgery of Trauma

Multivariate linear regression analysis for recovery showed that abdominal drainage was associated with a longer time to resume a soft diet and postoperative LOH. Moreover, a higher AAST grade was associated with a longer time to resume a soft diet and longer postoperative LOH (Table 4).

**Table 4:** Multivariate linear regression analysis for recovery parameters

	Time to resuming soft diet (hrs)		Postoperative length of stay (hrs)	
	β (95% CI)	*P* value	β (95% CI)	*P* value
*Drainage*		< 0.0001		< 0.0001
No	1		1	
Yes	20.697 (15.251–26.143)		45.899 (34.502–57.297)	
*Sex*		0.1749		0.2950
Female	1		1	
Male	2.859 (− 1.273 to 6.991)		4.618 (− 4.029 to 13.264)	
Age	0.114 (0.001–0.227)	0.0485	0.440 (0.203–0.677)	0.0003
AAST grade	6.874 (5.303–8.445)	< 0.0001	14.561 (11.273–17.848)	< 0.0001

AAST, The American Association for the Surgery of Trauma

The influence of drainage on complications was compared for each AAST grade ≥ III (Table 5). The drainage group was not associated with fewer IAA formation, but it was associated with higher overall complications and ileus in AAST grade IV + V and AAST grade IV + IV + V, than the non-drainage group (Table 5).

**Table 5:** Comparison of complications according to different grade of AAST grade ≥ III

	AAST V			AAST IV/V			AAST III/IV/V		
	-drainage (*N* = 51)	+ drainage (*N* = 68)	*P* value	-drainage (*N* = 131)	+ drainage (*N* = 140)	*P* value	-drainage (*N* = 177)	+ drainage (*N* = 171)	*P* value
*All complication, n (%)*			0.4007			**0.0129**			**0.0004**
Yes	15 (29.41)	25 (36.76)		28 (21.37)	49 (35.00)		34 (19.21)	62 (36.26)	
No	36 (70.59)	43 (63.24)		103 (78.63)	91 (65.00)		143 (80.79)	109 (63.74)	
*Clavien–Dindo classification, n (%)*			0.4142			0.7735			0.8847
I/II	13 (86.67)	19 (76.00)		23 (82.14)	38 (77.55)		27 (79.41)	50 (80.65)	
III/IV	2 (13.33)	6 (24.00)		5 (17.86)	11 (22.45)		7 (20.59)	12 (19.35)	
*Wound infection, n (%)*			0.9382			0.8414			0.6539
Yes	7 (13.73)	9 (13.24)		16 (12.21)	16 (11.43)		20 (11.30)	22 (12.87)	
No	44 (86.27)	59 (86.76)		115 (87.79)	124 (88.57)		157 (88.70)	149 (87.13)	
*Intra-abdominal abscess, n (%)*			> 0.9999			0.7507			> 0.9999
Yes	3 (5.88)	3 (4.41)		4 (3.05)	6 (4.29)		5 (2.82)	7 (4.09)	
No	48 (94.12)	65 (95.59)		127 (96.95)	134 (95.71)		172 (97.18)	164 (95.91)	
*Ileus, n (%)*			0.4253			0.0271			0.0013
Yes	5 (9.80)	10 (14.71)		8 (6.11)	20 (14.29)		10 (5.65)	28 (16.37)	
No	46 (90.20)	58 (85.29)		123 (93.89)	120 (85.71)		167 (94.35)	143 (83.63)	

AAST, The American Association for the Surgery of Trauma

## Discussion

The two key findings of this study are the following. First, patients with complicated appendicitis were not candidates for abdominal drainage. A higher AAST grade (≥ III) was not an indication for abdominal drainage, and abdominal drainage did not decrease the risks of overall complications or specific complications, such as IAA formation. Longer time to resume a soft diet and postoperative LOH were observed in patients with abdominal drainage. Therefore, abdominal drainage should be performed sparingly. Second, the algorithm developed in this study could validate the severity of acute appendicitis in terms of the likelihood of complications and postoperative convalescence. We believe that the algorithm can serve as a tool for determining the AAST grade of patients with appendicitis in a future retrospective study.

A literature review revealed that most retrospective studies did not detail how they determined a patient’s AAST grade [13, 17]. During the process of classifying the AAST grade of each patient in our study, ambiguity in the descriptions of surgical findings in operation notes was encountered and became one particular challenge. For example, the description “Ascites was turbid.” might refer to infectious or reactive ascites. In addition, the location and distribution of infectious ascites may influence the severity of appendicitis. All of these confounded the determination of AAST grades III–V. Therefore, throughout the algorithm, we explicitly detailed the allocation of AAST grades. According to the algorithm in our study, the key determinant of AAST grades III–V largely depended on the presence of ascites on CT. If the presence of ascites is more than two 5-mm slices in the helical CT, the possibility of AAST grade V was relatively high, and vice versa.

Our study showed that the AAST grade using our algorithm accurately predicted the incidence of overall complications and the length of postoperative convalescence, including the LOH and time to resume a soft diet. We thus believe that our algorithm is reliable and can be employed to define the AAST grade for our patients. Additionally, this allocation process enabled us to evaluate the necessity of abdominal drainage.

Our results confirmed that complicated appendicitis was associated with a high risk of IAA formation; however, complicated appendicitis was not an absolute indication for abdominal drainage. This finding seems paradoxical, but it makes sense when considered in light of the notion that the universal and prophylactic use of abdominal drainage would have a negative rather than positive effect. The results of this study discouraged the universal use of abdominal drainage for adult patients with complicated appendicitis undergoing appendectomy, even for patients with AAST V. Our results were in accordance with the recommendations of guidelines proposed by large surgical societies worldwide [12, 18]. Several explanations for this have been proposed. For example, kinking or obstruction of the drain may lead to drainage dysfunction, and the tip of the abdominal drain may fail to drain the space where the abscess is formed [6].

In our study, the frequency of IAA formation in complicated appendicitis (2.9%) was lower than that reported in previous studies[7, 8] and comparable to that reported in one study [17]. Most IAAs could be controlled with intravenous antibiotics and did not require postoperative surgical drainage for the IAA. In our previous study of 40 consecutive patients with complicated appendicitis who underwent single-incision LA without routine drainage, only one patient required postoperative CT-guided drainage [19].

Our study further indicated that patients with increased disease severity, as reflected by a higher AAST grade, would not benefit from abdominal drainage (Table 5). Furthermore, in the drainage group, increased complications such as wound infection and postoperative ileus, were evident when compared with the nondrainage group—this is consistent with the findings of a previous study [1, 7].

Drainage placement was an independent factor associated with delayed resumption of an oral soft diet and a longer LOH (Table 4). A possible reason for this could be that the surgeon may treat patients with drainage more conservatively; drainage tended to be performed in patients with a higher grade of acute appendicitis. Surgeons also may delay giving permission for oral intake until a patient’s abdominal condition improved, and therefore, abdominal drainage delays discharge because of the additional waiting time until the abdominal drain can be removed.

### Limitations

There are some limitations. First, as a retrospective study, one of its limitations is the potential bias in the selection of information included in the operation notes. Thus, the allocation of AAST grade may have been biased because of data in the operative notes. Because of the absence of an allocation algorithm in the literature, we developed an algorithm to overcome this limitation. Second, the investigator who categorized the AAST grade was not blinded to the patient outcomes. This could have led to a potential bias. However, we designed an algorithm to overcome these problems and attempted to clearly define the complications. Third, the clinical pathway varied among surgeons, resulting in differences in postoperative recovery and the time to drain removal. A well-designed, prospective randomized trial could compensate for the inadequacies of the retrospective analysis.

## Conclusion

Complicated appendicitis is a risk factor for IAA formation; however, it is not an absolute indication for abdominal drainage in patients undergoing LA. Abdominal drainage is not a mandatory procedure because it fails to prevent overall complications and specific complications such as IAA formation. Moreover, it is associated with prolonged gastrointestinal recovery and postoperative LOH. Our results indicate that the routine use of abdominal drainage should be discouraged and suggest that abdominal drainage should be performed sparingly.

## Data Availability

The datasets generated and analyzed during the current study are not publicly available due legal restrictions imposed by the government of Taiwan (R.O.C) on the distribution of the “Personal Information Protection Act”. But the datasets are available from the corresponding author on reasonable request.

## Abbreviations

LA: Laparoscopic appendectomy
AAST: American Association for the Surgery of Trauma
IAA: Intra-abdominal abscess
CT: Computed tomography
LOH: Length of hospitalization
MOST: Ministry of Science and Technology
